# Meningitis post-cochlear implant and role of vaccination

**DOI:** 10.15537/smj.2022.43.12.20220426

**Published:** 2022-12

**Authors:** Ghadah A. Alanazi, Aumniyat S. Alrashidi, Khalid S. Alqarni, Saeed A. Al khozym, Saad Alenzi

**Affiliations:** *From the Faculty of Medicine(Alanazi, Alrashidi), Tabuk University; from the Department of Otolaryngology (Alqarni, khozym, Alenzi), King Fahad Specialist Hospital, Tabuk, Kingdom of Saudi Arabia.*

**Keywords:** cochlear implant, bacterial meningitis, vaccine, otitis media

## Abstract

**Objectives:**

To investigate the incidence, risk factors, and management of meningitis in cochlear implant (CI)users.

**Methods:**

A systematic review was carried out using PubMed, Scopus, Web of Science, and Cochrane Central Register. Articles were considered relevant if reported any data on incidence, clinical presentations, the role of vaccination, management, and outcomes of meningitis after CI.

**Results:**

A total of 32 studies including 27358 patients were included, and meningitis was reported in only 202 cases. Meningitis occurred in the period ranging from 1 day to 72 months after CI. A total of 55 patients received the pneumococcal vaccine, while 20 patients received the *Haemophilus influenzae* type B vaccine. A large number of participants (n=47) had associated anatomical malformations, while 62 had otitis media before meningitis. A total of 24 cases required revision surgery along with medical treatment. Full recovery was the outcome reported by the included studies in 19 patients.

**Conclusion:**

Cochlear implant users seem to be at possible risk of bacterial meningitis at any time after implantation, especially in the presence of risk factors, such as otitis media and anatomical malformations of the cochlea.

**C**ochlear implants (CI) are commonly used interventions for children and adults with sensorineural hearing loss (SNHL). Cochlear implants utilisation among children began in 1980 and has been significantly increasing.^1^ The CI devices carry electrical stimulation using specific electrodes to the fibres of the cochlear nerve which provides an efficacious and reliable method of rehabilitation for patients with SNHL.^2,3^

Several adverse events may follow CI such as device malfunction, bleeding, and electrode migration, fascial nerve stimulation, and infection.^3^ It has been reported that CI may carry a higher risk of bacterial meningitis in comparison with the general population.^4-8^ In 2002, the United States Food and Drug Administration released a notification of receiving several reports, indicating a potential correlation between CI and the incidence of bacterial meningitis.^9^ Bacterial meningitis is a critical illness with remarkable morbidity and mortality rates. The most commonly identified meningitis organisms in a previous report were *Streptococcus pneumoniae (S. pneumonia*) and *Haemophilus influenzae*. The death rate from pneumococci-induced bacterial meningitis has been estimated to range from 15% to 60%.^10^

There is a lack of knowledge regarding the direct effects of meningitis on CI; however, some reports have mentioned the failure of implanted devices that require reimplantation. Several risk factors may contribute to meningitis in CI users, including otitis media, head trauma, cochlear malformations, and cerebrospinal fluid (CSF) leaks.^4-8^ The clinical features of post-CI meningitis do not differ from those of classical meningitis which include high temperature, headache, neck stiffness, photophobia, sickness, and vomiting. However, CI users may experience further complaints such as vertigo.^11^ It has been recommended that CI candidates and users should receive full immunization against organisms causing bacterial meningitis, especially *S. pneumoniae*, as it is more prevalent.^12,13^ The CDC recommendations for CI patients who never received pneumococcal vaccines include receiving of one shot of pneumococcal conjugate vaccine 15 followed by one shot of pneumococcal polysaccharide vaccine 23.^14^

There is a lack of systematic reviews that assemble published reports on meningitis among CI users and draw comprehensive recommendations or conclusions. This systematic review aimed to report meningitis occurrence among CI users in terms of patients’ demographic characteristics, incidence, associated risk factors, possible immunization role, and management.

## Methods

A systematic literature search was carried out following the Preferred Reporting Items for Systematic Reviews and Meta-Analysis recommendations. The project protocol was written and registered in PROSPERO (CRD42021288471).

In November 2021, a systematic search was conducted in PubMed, Scopus, Web of Science, and Cochrane Central Register using these search terms (Meningitis AND [CI OR cochlear implantation]). We aimed to retrieve all search results without applying any search filters. All search results were combined into one Endnote library, and all duplicated references were removed. Articles were then transferred to an Excel spreadsheet for title/abstract screening for potential relevance. Articles were considered relevant if they were original studies reporting any data on the incidence, clinical presentations, role of vaccination, management, and outcomes of meningitis after cochlear implantation. There were no restrictions on the year or place of research, patient age, or study design. However, we did not include i) conference papers, comments, letters, review papers, or book chapters; ii) articles with overlapping data sets; iii) non-English articles; and iv) animal studies. Two authors independently screened articles according to the aforementioned criteria. Further screening rounds using full texts were also performed for the final decision on the inclusion or exclusion of specific reports.

Three reviewers independently extracted the data from the final full texts. Extracted data included information on individuals’ demographic characteristics, sample size, the number of CI users with meningitis, and implant device type. It also comprises data on the number of episodes, causative organism, risk factors, immunization status, diagnostic method, management plan, and outcome. Any discrepancies were resolved through discussion with a fourth author.

### Statistical analysis

Descriptive analysis of the extracted data was carried out using Microsoft Excel to estimate the numbers, percentages and means.

## Results

**Figure 1:**
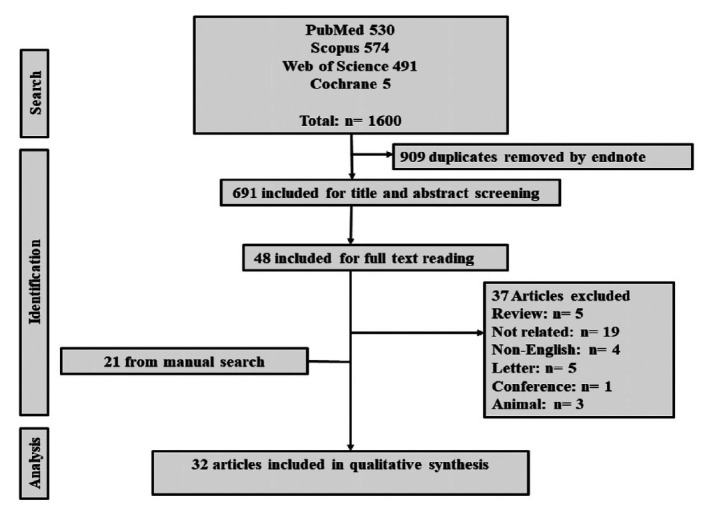
- Flow diagram of studies selection and screening.

A total of 1600 reports were obtained from the databases, of which 909 were removed as duplicate references. After title/abstract screening, only 48 remained for full-texts review. Manual reference checks retrieved more reports; eventually, 32 articles were eligible for analysis (Figure 1).

A total of 32 studies published between 1991 and 2020 and 27358 patients were included.^4-6,8,10,14-40^ Seventeen were case studies while 15 were retrospective observational studies. Most of the studies (37.5%) were carried out in the United States. The mean age of the patients with meningitis after CI ranged from 1 to 11 years. Among the 23 studies reporting patient gender, there were 45 (64.3%) male patients and 25 (35.7%) female patients. The type of implant device and the basic demographic characteristics are detailed in Table 1.

**Table 1:** - Baseline demographic characters of enrolled studies.

Author/year	Country	Design	Total sample size	Meningitis patients (N)	Age mean Y (Range)	Male/Female	Device type
Cohen & Hoffman^15^/1991	USA	ROS	921	23	-	-	(23) Nucleus device
Daspit^6^/1991	USA	Case report	1	1	51	Male	(1) Nucleus device
Webb et al^16^/1991	Germany	ROS	712	1	-	-	(1) multichannel implant
Page & Eby^17^/1997	UK	Case report	1	1	5	Male	-
Woolley et al^18^/1998	USA	Case series	4	1	5 y 4 months	Female	(1) multichannel implant
Reefhuis etl al^19^/2003	USA	POS	4264	26	(11-6)	18/8	(4) CI24M, (11) AB-5100H, (2) AB-5100,(7) AB-5100H-11, (2) CI24RST, (2) CI22M, (1)C40+HS
Callanan & Poje^10^/2004	USA	Case series	2	2	3-5	Male	(2) Clarion C-II device with positioner
Pettersen^20^/2005	Canada	Case report	1	1	4	Male	(1) Advanced bionics with positioner
Biernath KR^21^/2006	USA	ROS	4265	12	(21-103) months	9/3	(8) AB-5100H-11, (1) C40HGB, (3) AB-5100H
Ahn et al^22^/2008	Korea	ROS	388	1	5.2	Male	(1) HF.1.2
Mancini et al^23^/2008	Italy	Case series	3	3	(9-11)	2/1	(2) Clarion 1.2, 1 Clarion HiFocus CII
Torkos et al^24^/2009	Hungary	Case report	1	1	2	Male	(1) Nucleus 24 R
Loundon et al^25^/2010	France	ROS	434	1	6	-	-
McJunkin & Jeyakumar^26^/2010	USA	ROS	165	1	5	Female	(1) Med El
Ahn et al^27^/2011	Korea	ROS	11	2	-	1/1	(1) HF 1.2, (1) CI24RST
Ajallouyean et al^4^/2011	Iran	Case series	262	1	1.5	-	-
Gurbuz et al^7^/2011	Turkey	Case report	1	1	8.5	Female	(1) Med-El© short electrode
Lalwani & Cohen^28^/2011	USA	ROS	8,329	86	43 <5 y	-	(86) devices with positioner
O’Mahony et al^29^/2011	USA	Case report	1	1	4	Male	(1) Implant with positioner
Mancini et al^8^/2013	Italy	Case series	5	5	(2.5-8)	2/3	AB devices: 2 with Clarion 1.2 HiFocus with positioner, 1 with HiFocus CII and 1 with HiResolutionTM 90K; 1 with a Med-El Combi 40+
Roman et al^30^/2013	USA	Case report	1	1	2	Female	(1) Nucleus CI24 straight electrode array
Tarkan et al^31^/2013	Turkey	ROS	475	2	1.5-2.5	-	(1) Med-El Sonata (straight array), (1) Nucleus CI24RE (contour array)
Afsharpaiman et al^32^/2014	Iran	ROS	371	1	24 months	Female	-
Suleiman et al^33^/2014	Nigeria	Case series	2	1	8	Female	(1) Medel Pulsar
Daneshi et al^34^/2015	Iran	ROS	4346	4	-	-	-
Javia et al^35^/2016	USA	ROS	478	4	(1.3-4)	3/1	(1) Clarion CII, 1 Clarion HiFocus, 2 AB HiRes 90K
Pross et al^36^/2016	USA	Case report	1	1	4	Female	-
Tandon et al^37^/2016	India	case report	1	1	1	Male	(1) Nucleus freedom implant CI24RE (straight electrode) with straight electrode array
Vartanyan et al^38^/2018	Australia	Case report	1	1	23	Female	-
Dağkıran et al^5^/2020	Turkey	ROS	1452	5	-	-	-
Yan et al^39^/2020	China	Case report	1	1	7	Male	-
Piromchai et al40/2021	Thailand	ROS	458	9	-	-	(9) 3M device

ROS: retrospective study, POS: prospective study, USA: United Stated of America, Y: years, AB: advanced Bionics, HS: Houston Galveston Brazoria, HF: HiFocus electrode array

### Incidence of meningitis and vaccination status

Of the 27358 who performed CI, 202 (0.7%) who developed menigitis were enrolled in the study. Meningitis occurred in the period ranging from 1 day to 72 months after CI. *Streptococcus* *pneumoniae* was responsible for the incidence of meningitis in 42 episodes of infection. Ten patients experienced more than one episode of meningitis. A total of 55 patients had a history of immunization with the pneumococcal vaccine, while 20 patients received the *Haemophilus influenzae* type B vaccine (HIB). The remaining studies did not report any detailed data regarding the pathonomic organism or previous vaccination. Regarding potential risk factors, as per the authors’ report, a large number of participants (n=47) had associated anatomical malformations. Moreover, 62 patients had otitis media before meningitis and 23 had a history of CSF leak after CI (Table 2).

**Table 2:** - Reported meningitis with associated risk factors and role of vaccination.

Author/year	Meningitis cases (N)	Onset of meningitis (month)	Organism	N. of meningitis episodes	Immunization status	Possible risk factors			
						Anatomical deformities N of patients	CSF leak	Otitis media	Others
Cohen & Hoffman^15^/1991	23	-	-	-	-	-	-		-
Daspit^6^/1991	1	0.2	*Streptococcus pneumonia*	-	-	-	1		Head trauma and bilateral Meniere’s disease
Webb et al^16^/1991	1	-	-	-	-	-	-		-
Page & Eby^17^/1997	1	24	-	2	-	1	-		Sinusitis prior to meningitis onset
Woolley et al^18^/1998	1	7	-	1	-	1	1	1	
Reefhuis et al^19^/2003	26	1 day-36 months	*Streptococcus pneumoniae*: 15 episodes *Haemophilus influenzae* type b: 5 episodes Acinetobacter baumannii: 2 episodes Enterococcus: 1 episode, *Escherichia coli*: 1 episode, Unknown: 5 episodes	23 patient: 1 3 patients: 2	-	9	6	13	Previous meningitis: 5 ventriculoperitoneal shunt: 1
Callanan & Poje^10^/2004	2	0.4-12	*Haemophilus influenzae*: 1 patient *Streptococcus pneumoniae*: 1 patient	1	HIB and the pneumococcal vaccines	1	1	1	
Pettersen et al^20^/2005	1	42	Group A streptococcus: 1st episode, *Pseudomonas aeruginosa*: 2^nd^ episode	2	Pneumococcal and Neisseria meningitidis vaccines	-	-	2	
Biernath et al^21^/2006	12	5-40	*Streptococcus pneumoniae*: 9 patients Group A Streptococcus: one patient Unknown: 2 patients	1	-	2	1		Previous meningitis: 3
Ahn et al^22^/2008	1	-	-	3	-	1	-		-
Mancini et al^23^/2008	3	8-72	*Streptococcus pneumoniae*: 3 episodes, Negative CSF: one episode	2 patients: 1 1 patient: 2	Pneumococcal vaccine	2	-		-
Torkos et al^24^/2009	1	6	*Haemophilus influenzae* in episode, *Streptococcus pneumoniae* in another	4	-	1	1		-
McJunkin & Jeyakumar^26^/2010	1	12	*Pseudomonas aeruginosa*	1	-	1	-		-
Loundon et al^25^/2010	1	48	*Streptococcus pneumoniae*	1	Complete immunization history	-	-		-
Ahn et al^27^/2011	2	48	-	-	-	2	1		-
Ajallouyean et al^4^/2011	1	-	-	-		-	-		-
Gurbuz et al^7^/2011	1	24	*Streptococcus pneumonia*	4	HIB vaccine before first 3 episodes. Pneumococcal vaccine before the last episode	1	1		Head trauma

HIB: Haemophiles influenzae type B, CSF: cerebrospinal fluid

**Table 2:** - Reported meningitis with associated risk factors and role of vaccination (continuation).

Author/year	Meningitis cases (N)	Onset of meningitis (month)	Organism	N. of meningitis episodes	Immunization status	Possible risk factors			
						Anatomical deformities N of patients	CSF leak	Otitis media	Others
Lalwani Cohen^28^/2011	86	59 within the first 24 month	-	-	Fully immunized: 31 HIB vaccine: 11 Not immunized: 22 Unknown: 22	9	-	42	Previous meningitis: 7
O’Mahony et al^29^/2011	1	36	Streptococcus pneumoniae	1	Complete immunization history	1	-		-
Mancini et al^8^/2013	5	3 -72	Streptococcus pneumoniae	-	Pneumococcal vaccine	3	-	4	
Roman et al^30^/2013	1	5-10	Streptococcus pneumoniae in the first episode, Haemophilus influenza in the 2nd episode	2	HIB and the pneumococcal vaccines	1	1		-
Tarkan et al^31^/2013	2	1, 30	-	1 patient: 3	Pneumococcus vaccine	2	1		-
Afsharpaiman et al^32^/2014	1	-	-	-	Complete immunization history but not with Pneumococcal vaccine	-	-		-
Suleiman et al^33^/2014	1	-	-	1	Pneumococcal vaccine	0	-		Previous meningitis
Daneshi et al^34^/2015	4		-	-	Pneumococcal vaccine				
Javia et al^35^/2016	4	4 days-32 months	Acinetobacter baumannii: one patient, Streptococcus pneumoniae: one patient, Negative CSF: 2 patients	1	-	-	1		-
Pross et al^36^/2016	1	3	Streptococcus pneumoniae	2	Pneumococcal vaccine	1	-		Previous meningitis
Tandon et al^37^/2016	1	4-13	Nonhemolytic streptococci: 1st episode, Streptococcus pneumoniae: 2nd episode	3	Pneumococcal, meningococcal, and HIB vaccines	1	1		Respiratory tract infection prior to meningitis
Vartanyan et al^38^/2018	1	72	Streptococcus pneumoniae	1	HIB and Neisseria meningitides vaccines	1	1		-
Dağkıran et al^5^/2020	5	-	-	-	-	5	4		-
Yan et al^39^/2020	1	72	Pseudomonas aeruginosa	1	-	1	1		Head trauma
Piromchai et al^40^/2021	9	-	-	-	-	-	-		-

HIB: Haemophiles influenzae type B, CSF: cerebrospinal fluid, N: number

### Management and outcomes of meningitis cases after CI

Clinical evaluation and CSF culture were frequently reported methods of diagnosis (n=18 studies); otherwise, they were not reported. A total of 42 infection episodes were managed only through medical treatment, whereas 24 episodes required revision surgery along with medical treatment. Full recovery was the outcome reported by 19 patients in the included studies (Table 3).

**Table 3:** - Management and outcomes for patients with meningitis after cochlear implant.

Author/year	N. of patients had meningitis	Diagnostic method	Management	Outcomes
Cohen & Hoffman^15^/1991	23	-	-	-
Daspit^6^/1991	1	Clinical+ CSF culture	Medical treatment	Full-recovery
Webb et al^16^/1991	1	-	-	Full-recovery
Page & Eby^17^/1997	1	-	1st episode medical treatment, 2nd episode Revision surgery + medical treatment	Quadriparesis
Woolley et al^18^/1998	1	Clinical+ CSF culture	Revision surgery + medical treatment	Full-recovery
Reefhuis et al^19^/2003	26	Clinical+ CSF culture	3 Revision surgery + medical treatment, 23 medical treatment	1 Died, 3 implant removed
Callanan & Poje^10^/2004	2	Clinical+ CSF culture	Medical treatment	Full-recovery
Pettersen et al^20^/2005	1	Clinical+ CSF culture	1st episode medical treatment, 2nd episode Revision surgery + medical treatment	Language delay with loss of his milestones
Biernath et al^21^/2006	12	Clinical+ CSF culture	-	2 died
Ahn et al^22^/2008	1	-	-	Re-implantation was required
Mancini et al^23^/2008	3	Clinical+ CSF culture	3 Medical treatment, 1 medical treatment+ revision surgery in the second episode	2 Fibrosed cocclea require reimplantation,1 full recovery
Torkos et al^24^/2009	1	Clinical+ CSF culture	1 Revision surgery + medical treatment	Full-recovery
McJunkin & Jeyakumar^26^/2010	1	Clinical+ CSF culture	-	-
Loundon et al^25^/2010	1	Clinical+ CSF culture	Medical treatment	Full-recovery
Ahn et al^27^/2011	2	-	1 Revision surgery + medical treatment, 1 medical treatment	-
Ajallouyean et al^4^/2011	1	-	-	-
Gurbuz et al^7^/2011	1	Clinical+ CSF culture	The first 3 episodes were treated with medical treatment and the last episode treated with revision surgery + medical treatment	-
O’Mahony et al^29^/2011	1	Clinical+ CSF culture	medical treatment+ shunt surgery	Right hemiparesis
Lalwani & Cohen^28^/2011	86	-	-	-
Mancini et al^8^/2013	5	-	2 Revision surgery + medical treatment	2 ipsilateral ossification, 1ipsilateral and contralateral ossification
Roman et al^30^/2013	1	Clinical+ CSF culture	1 Revision surgery + medical treatment	Full-recovery
Tarkan et al^31^/2013	2	-	1 Revision surgery + medical treatment, 1 medical treatment	Full-recovery
Afsharpaiman et al^32^/2014	1	-	-	-
Suleiman et al^33^/2014	1	-	Medical treatment	Full-recovery
Daneshi et al^34^/2015	4			
Pross et al^36^/2016	1	Clinical+ CSF culture	1 Medical treatment	Full-recovery
Javia et al^35^/2016	4	Clinical+ CSF culture	2 Revision surgery + medical treatment, 2 medical treatment	1 Temporary hemiparesis that resolved, 3 Full-recovery
Tandon et al^37^/2016	1	Clinical+ CSF culture	The first 2 episodes medical treatment, the 3rd episode revision surgery + medical treatment	Full-recovery
Vartanyan et al^38^/2018	1	Clinical+ CSF culture	1 Revision surgery + medical treatment	Full-recovery
Dağkıran et al^5^/2020	5	-	4 Revision surgery + medical treatment, 1 medical treatment	-
Yan et al^39^/2020	1	Clinical+ CSF culture	1 Revision surgery + medical treatment	Full-recovery
Piromchai et al^40^/2021	9	-	-	-

CSF: cerebrospinal fluid

## Discussion

This review investigated the incidence of meningitis in CI users. The incidence has been reported in a minority of cases (0.7%) involving several risk factors. Meningitis may occur at any time point after CI, which warrants the immediate identification of suspected cases for earlier management.^41^ The exact mechanism underlying the incidence of meningitis after CI remains unclear. Probable mechanisms have been previously investigated in animal research that suggested that immune response, by way or another, is affected by the presence of the foreign body in the inner ear.^12^ Our study showed that some patients who were vaccinated against meningitis may still have been infected. Vaccination against bacterial meningitis in developed nations has become routine practice. Moreover, it is recommended that children undergoing surgical interventions have an increased risk of post-surgery meningitis. Owing to the vast number of immunization practices, the incidence of meningitis has declined significantly, and such a reduction has been reported in European and North American countries.^42,43^ In contrast, immunization against meningitis in developing countries is still challenging and carries a financial burden as being not supported by their local healthcare systems.^32^ Regarding children who are CI recipients or scheduled for CI, conjugate vaccines against *S. pneumoniae* and *H. influenzae* are recommended for all patients with CI, while the meningococcal vaccine is not regularly required.^41,44,45^ Moreover, in children aged 2-9 years, a conjugate pneumococcal vaccine followed by a 23-valent pneumococcal polysaccharide vaccine is recommended. Therefore, healthcare workers should check immunization records to ensure that all the required vaccines have been administered.^20,45^

A large number of patients in the present study had otitis media before meningitis. As *S. pneumoniae* and *H. influenzae* are commonly identified organisms in the CSF, it is often proposed that these organisms take the route of the middle ear to the cochlea and eventually to the meninges.^46^ However, clinical and animal studies have reported that both implanted and non-implanted cochlea can withstand the spread of infection with similar effectiveness.^47^ Nonetheless, CI doctors should discuss with infectious disease physicians regarding the optimal treatment for otitis media and its complications. Patients with CI who present with features of acute otitis media should be managed urgently to prevent the transmission of infection to the cochlea. Beside prophylaxis antibiotics for CI users who are known to be otitis-prone, immediate identification and aggressive management of each episode of acute otitis media should involve tympanostomy tubes and full course of antibiotic.^44^ Moreover, patients undergoing CI with known cochlear malformations, such as Mondini, and enlarged vestibular aqueduct, are considered vulnerable to the risk of otogenic meningitis.^48^

Our study showed that most infection episodes were managed with medical treatment alone. Parents of children with CI must be able to recognize the potential clinical features and seek medical evaluation immediately if their child’s hearing is altered. The recommended empirical antibiotics for the management of patients with CI with suspected meningitis do not differ from the routine recommendations for other cases of meningitis, as the organisms are similar. The current treatment regimen should include vancomycin combined with a broad-spectrum cephalosporin. In cases of chronic otorrhea or perforated tympanic membranes, antibiotics such as ceftazidime, cefepime, or meropenem are recommended to fight probable *P. aeruginosa*.^46,49^ Of note, antibiotics must be chosen based on the identified microorganism once CSF infection is evident; however, the course length may vary owing to a lack of consensus in the literature.^20^

### Study limitations

First is the small sample size of the included studies, with approximately half of them being case studies. However, we searched for relevant reports using four major databases, along with the manual search of relevant reference lists. Second, there is a lack of meta-analyses owing to the limited number of studies and data heterogeneity. Immunization status has not been discussed in some studies. We believe that the findings of our study should be cautiously interpreted, and future studies are recommended to further evaluate the role of vaccination and identify potential risk factors. Public health workers and

should provide proper knowledge and awareness to primary care health practitioners and family members of children with CI regarding the early symptoms of otitis media and meningitis. Thus, antibiotics can be initiated immediately once the symptoms appear.^41,46^ They should be aware of the possible bacterial invasion from the middle ear to the meninges. At all times and taking all precautions, CI candidates and their close members should be aware of the continuous risk of post-implant meningitis before implantation. Future experimental studies are recommended to evaluate the exact role of meningitis vaccination in minimizing the risk of infection or complications. Moreover, data from large multicentre studies are recommended to establish generalized measures.

In conclusion, given the current findings from the systematic literature search, CI users seem to be at possible risk of bacterial meningitis at any time after implantation. Specific factors may increase the risk of infection, such as otitis media, anatomical malformations of the cochlea, and CSF leak. Primary care physicians and family members of CI users should be aware of the presenting features of meningitis and otitis media. There are no different management guidelines for meningitis in CI users; therefore, the key element in curbing the risk is extending CI-specific vaccination campaigns to be followed and recommended. CI candidates should be educated regarding the possible risk of post-implant meningitis, particularly if they have any of the potential risk factors.
